# Phenanthroindolizidine Alkaloids Secondary Metabolites Diversity in Medicinally Viable Plants of the Genus *Tylophora*

**DOI:** 10.3390/plants12051143

**Published:** 2023-03-02

**Authors:** Ehab M. Mostafa, Arafa Musa, Hamdoon A. Mohammed, Abdulaziz Ibrahim Alzarea, Mohamed A. Abdelgawad, Mohammad M. Al-Sanea, Ahmed Ismail, Ameeduzzafar Zafar, Mohammed Elmowafy, Samy Selim, Riaz A. Khan

**Affiliations:** 1 Department of Pharmacognosy, College of Pharmacy, Jouf University, Sakaka 72341, Saudi Arabia; 2 Department of Medicinal Chemistry and Pharmacognosy, College of Pharmacy, Qassim University, Buraydah 51452, Saudi Arabia; 3 Pharmacognosy and Medicinal Plants Department, Faculty of Pharmacy (Boys), Al-Azhar University, Cairo 11884, Egypt; 4 Department of Clinical Pharmacy, College of Pharmacy, Jouf University, Sakaka 72341, Saudi Arabia; 5 Department of Pharmaceutical Chemistry, College of Pharmacy, Jouf University, Sakaka 72341, Saudi Arabia; 6 Pharmacognosy Department, Faculty of Pharmacy, Fayoum University, Faiyum 63514, Egypt; 7 Department of Pharmaceutics, College of Pharmacy, Jouf University, Sakaka 72341, Saudi Arabia; 8 Department of Clinical Laboratory Sciences, College of Applied Medical Sciences, Jouf University, Sakaka 72388, Saudi Arabia

**Keywords:** genus *Tylophora*, phenanthroindolizidine alkaloids, chemotaxonomy, secondary metabolites, anticancer, antiviral, antimicrobial, anti-inflammatory, phytochemicals, biological activities

## Abstract

Plants of the genus *Tylophora* have commonly been used in traditional medicine in various communities, especially in the tropical and subtropical regions of climatic zones. Of the nearly 300 species reported in the *Tylophora* genus, eight are primarily used in various forms to treat a variety of bodily disorders based on the symptoms. Certain plants from the genus have found use as anti-inflammatory, anti-tumor, anti-allergic, anti-microbial, hypoglycemic, hypolipidemic, anti-oxidant, smooth muscle relaxant, immunomodulatory, and anti-plasmodium agents, as well as free-radical scavengers. Pharmacologically, a few plant species from the genus have exhibited broad-spectrum anti-microbial and anti-cancer activity, which has been proven through experimental evaluations. Some of the plants in the genus have also helped in alcohol-induced anxiety amelioration and myocardial damage repair. The plants belonging to the genus have also shown diuretic, anti-asthmatic, and hepato-protective activities. *Tylophora* plants have afforded diverse structural bases for secondary metabolites, mainly belonging to phenanthroindolizidine alkaloids, which have been found to treat several diseases with promising pharmacological activity levels. This review encompasses information on various *Tylophora* species, their distribution, corresponding plant synonyms, and chemical diversity of the secondary metabolic phytochemicals as reported in the literature, together with their prominent biological activities.

## 1. Introduction

Natural products are of great ecological and functional importance. They are primarily used as medicines for large populations around the world, as well as in conjunction with modern medicine for various illnesses, particularly various cancer forms [1,2]. Their role as reservoirs of new structural templates, which are plentifully available in nature as part of the secondary metabolic products of the plants, has further increased their role and demands. The contributions of plant-based products, including crude drugs, have provided raw materials to manufacturers. Plant products are important on an everyday basis and constitute the principal blocks for drug development, discovery, and structural template modifications [3,4,5,6]. They have also piqued the interest of researchers looking to solve new and recurring global health issues such as infectious and non-communicable diseases. In that context, several thousands of the plants have been phytochemically examined and reported for their biological activities and structural variations [7,8]. Continuous efforts of the researchers have afforded several natural product-based modern drugs that are in use today for curing and protecting against diseases. The researchers have also discovered thousands of natural products from plants, which have been categorized into several classes, including phenolics, flavonoids, alkaloids, glycosides, iridoids, saponins, volatile oils, and bitter principles. Alkaloids are a class of natural products that have piqued the interest of scientists around the world due to their potent activities and therapeutic potential [9,10]. Several of these alkaloids are available on the modern drug market and are used to treat a variety of diseases, including cancer (Vinca and Taxus origin alkaloids and the phenanthroindolizidine structural class of alkaloids), gastrointestinal disorders (tropane alkaloids), hypnotic effects (coca alkaloids), analgesia (morphinans), malaria (cinchona alkaloids), oxytocic conditions (ergometrine), and disorders of the central nervous system. This review discusses the phenanthroindolizidine alkaloids-rich plant genus, *Tylophora*, and the diversity of the alkaloids and other secondary metabolites in plants of this genus, along with their biological activities and established pharmacological effects. 

*Tylophora* (family Asclepiadaceae) is widely distributed, primarily in Australia, Asia, and Africa [11,12]. The name *Tylophora* comes from the Greek words “tylos”, which means knot, and “phore”, which means carrier or bearer. *Tylophora indica* is the most common species in this genus and is used in most traditional medicines. It is an annual and perennial, small, slender, climbing, and much-branched young herb [13]. It has been reported that plants from this genus are used in traditional medicine for treating bronchial asthma, rheumatism, allergies, and dermatitis [14,15,16,17]. In addition, the plant has anti-tumor, anti-oxidant, immunomodulatory, and hypotensive activities. It is also used for treating numerous respiratory difficulties, such as asthma, bronchitis, hay fever, the common cold, and coughs [18,19,20]. Moreover, the leaves and roots of this plant have been used to treat jaundice and symptomatic liver disorders [14,21,22,23]. *Tylophora* genus plants also possess a wide range of bioactivities, including immune modulatory effects, free-radical scavenging (anti-oxidant), hepato-protective, anti-convulsant [14,24,25,26], anti-anxiety, anti-bacterial, anti-asthmatic, anti-inflammatory, anti-cancer, anti-amoebic [14,16,20,26,27,28,29], anti-psoriasis, seborrhic, and anaphylactic effects, and they are used for the treatment of leucopenia and the Schultz-Dale reaction. The roots and leaves of the plant, *Tylophora indica*, possess medicinal and therapeutic properties as an expectorant, laxative, diaphoretic, and purgative [30,31,32]. Several secondary metabolites from *Tylophora* species have been isolated, including alkaloids such as tylophorine, tylophorinidine, septicine, and tylophorine, as well as non-alkaloidal compounds such as quercetin, tannin(s), tetratriacontanol, α- and β-amyrins, octaosanyl octacosane [20,33,34,35,36,37]. Tylophorinine and tylophorine are the main alkaloids found in this genus, and have been shown to be responsible for the strong anti-inflammatory effects exhibited by plants in the genus [38,39]. The phenanthroindolizidine class of alkaloids, isolated and identified as the tylophorine class, is exemplified and represented by tylophorinidine, tylophorine, tyloindicines A–J, tylocrebrine, tylophorinine, and *O*-methyl tylophorinidine, as active components. These alkaloids are known to possess potent anti-cancer activity through participation different mechanisms of action [40,41,42]. 

The current review surveys the literature retrieved from various search engines, including PubMed^®^, ScienceDirect^®^, Scopus^®^, and Google Scholar^®^, and provides information on the geographic distribution of the genus *Tylophora*’s, the occurrence and frequency of the plant’s alkaloidal constituents, their biogenesis, and the alkaloids’ roles in bioactivities of different kinds. Information on isolation-extraction procedures, yields, and plant enrichment status of the phenanthroindolizidine alkaloidal constituents, the alkaloids’ chemical structures and their chemo-structural diversity of substituents and ring structures, as well as the inputs from the structural diversity of these recognized secondary metabolites corresponding to the plant sources, and their medicinal uses, is discussed. The review also contains details on the plants’ uses as part of traditional phytomedicines by locals in different geographical areas, and the scope of these alkaloids in modern medicine, including their roles in contemporary drug design, discovery, and development, especially in anti-cancer and anti-inflammatory pharmacological classes. The review focuses on the medicinally viable plants of the *Tylophora* genus and identifies gaps for future research. The review also highlights the potential applications of phenanthroindolizidine alkaloid compounds in the development of new therapies based on their biological activity, and provides, to a certain extent, the rationale for their traditional phytomedicinal uses in different communities.

## 2. Taxonomy of Genus *Tylophora*

### 2.1. Geographic Distribution

*Tylophora* plants are widely distributed in the southern hemisphere and some smaller regions in the north, where warm and wet climates are found. These perennial climbers are native to forests, grasslands, and hills in the southern and eastern Indian subcontinent, as well as other parts of Asia, including Sri Lanka, Malaysia, Thailand, Australia, and the Pacific Islands. Although the genus did not originate there, the plants are also found on the African continent. Over 300 species of *Tylophora* have been identified and recorded to date. These plants belong to the division Angiosperm, class Mangoliatae, order Gentianales, family Asclepidaceae, and genus *Tylophora* [34,43,44,45].

### 2.2. Morphological Description

The plants in the genus are mostly erect herbs or woody climbers, but some of the species are succulents. The leaves are simple, sub-sessile, exstipulate, fleshy, and covered with a coating of wax. Flowers are cymose, or racemose inflorescence, pedicellate, bracteolate, bisexual, and actinomorphic, but rarely zygomorphic. Five polysepalous sepals and five gamopetalous petals have been described. The androecium is composed of five stamens, and the anthers are syngenesious, giving rise to a five-sided blunt cone, which is usually attached on the inside to the stigma head. In the gynoecium, which is bicarpellary, the ovaries remain free, but styles unite to form a commonly swollen stigma-head. The placentation is marginal, with numerous ovules. Fruits are produced by pairs of follicles, but sometimes there is only one follicle because of the suppression of the other. The seeds are ovate-oblong, flat, and capped by hairs or fruits, and these hairs enable the seeds to be dispersed by the wind. The embryo is large [46]. *Tylophora* indica (Burm f.) Merill., also known as Anantmool in the vernacular language, is a perennial plant found in southern and eastern India in plains, forests, and hilly areas. The climbing shrub, or twining plant can reach up to 1.5 m in height, and is widespread in the provinces of Uttar Pradesh, Bengal, Assam, Orissa, and the Himalayan regions. The leaves are obovate-oblong to elliptic-oblong, and measure between 3–10 cm in length and 1.5–7 cm in width. The roots are long and fleshy, with a light brown, fissured corky bark. The flowers are small, about 1–1.5 cm across, and arranged in 2–3-flowered clusters in axillary umbellate cymes. The calyx is divided nearly to the base, with densely hairy lanceolate segments. The corolla is greenish-yellow, or greenish-purple, with oblong, acute lobes. The fruit is an ovoid-lanceolate follicle with 0.6–0.8 × 0.3–0.4 cm long seeds that taper at the apex, forming a fine mucro before becoming striate and glabrous [47].

A partial list of species in the genus *Tylophora* [48], and photographs of a number of these plants are illustrated in Table 1.

## 3. Traditional Phytomedicinal Uses of Genus *Tylophora* Plants

Plants belonging to the genus *Tylophora* have been extensively used in traditional phytomedicine. The aerial parts are taken orally for the treatment of constipation, flatulence, hemorrhoids, whooping cough, asthma, congestion, inflamed skin, jaundice, gout, tender joints, and arthritis. Their use also induces sweating and vomiting. *Tylophora* plants have also been applied topically for allergies, inflamed skin, wounds, and skin ulcers, and used as a smooth muscle relaxant, and anti-lupus agent [49]. The plants have been recommended by an informed herbalist for cancer and rheumatoid arthritis. *Tylophora indica* has been extensively used as a remedy for bronchial asthma, and to relieve mild pain, and dermatitis [50]. *T. indica* has been used in Pakistan to treat skin and systemic allergies, as well as bronchitis. It has also found use as an emetic, laxative, cathartic, purgative, stimulant, and diaphoretic in areas of Pakistan [24]. *T. indica* leaves have been used to treat tuberculosis and as an antidote to snake bites [51,52]. The plant has also found use as a muscle relaxant, an anthelmintic, for the treatment of hydrophobia, and as a food preservative [14,53]. Table 2 summarizes the ethnomedicinal uses of the *Tylophora* species found in different geographic locations and their local uses.

## 4. Chemotaxonomy of the Genus *Tylophora,* Phenanthroindolizidine, and Secophenanthroindolizidine Alkaloids

Chemotaxonomy, also known as chemosystematics, differentiates plant species based on chemical constituents with respect to their phenotypic category and specifications of biogenetically derived constituents, which are frequently of a secondary metabolic nature. This information has led to insights into the taxonomy of the plants, and has, in turn, has helped in metabolomics (metabolic profiling) understanding. In conjunction with morphological and cytological data, the array of chemical constituents of certain and defined structural features, found primarily in plant species within the genus, has intertwined the taxonomic classification and helped in plant identification and taxonomical classification. Genomics, transcriptomics, and proteomics relate the phenotype of a taxon to its genome, and this further strengthens the phenotypical characteristics to chemosystematics, thereby providing a foundation for the taxonomy and genomics. Chemogenomic systematics ignores the presence of small molecules in plants, which are frequently linked to environmental responses as well as biodiversity. From the perspective of chemical constituents, the presence of specific categories of compounds, and distinguishing secondary metabolic products, provide taxonomic factors, assist in testing their congruence with existing classifications to identify the chemotype, and at times predict the formation of inter-related secondary metabolites useful to human health and drug discovery. 

As structured entities, phenanthroindolizidine alkaloids are composed of a dibenzo-[f,h]pyrrolo [1,2-b]isoquinoline ring as the core structural motif in various alkaloids from *Tylophora* species and have thus been classified as chemotaxonomic or chemosystematic markers for the genus. However, interconnected structures have been observed in several species of the plant family Moraceae [71,72]. These alkaloidal structures are the focus of added attention, in addition to their diverse range of bioactivities and traditional uses, due to their anti-leukemic and other anti-cancer properties.

Phenanthroindolizidine alkaloids presence has been recorded among almost all 300 species of the genus *Tylophora*, which is part of the Asclepiadaceae family. Phenanthroindolizidine alkaloids are abundant in several species from four other genera besides *Tylophora*, namely *Pergularza, Cyanchum, Antitoxzcum*, and the genus *Vincetoxzcum* [71]. However, the alkaloid type has only been reported in one species of the Moraceae family, Ficus [72]. Examples of different phenanthroindolizidine and *seco*-phenanthroindolizidine (seco, or broken ring and rearranged) alkaloids isolated from the genus are listed below (Table 3 and Table 4).

## 5. Biogenesis of Genus *Tylophora* Alkaloids

**Figure 1:**
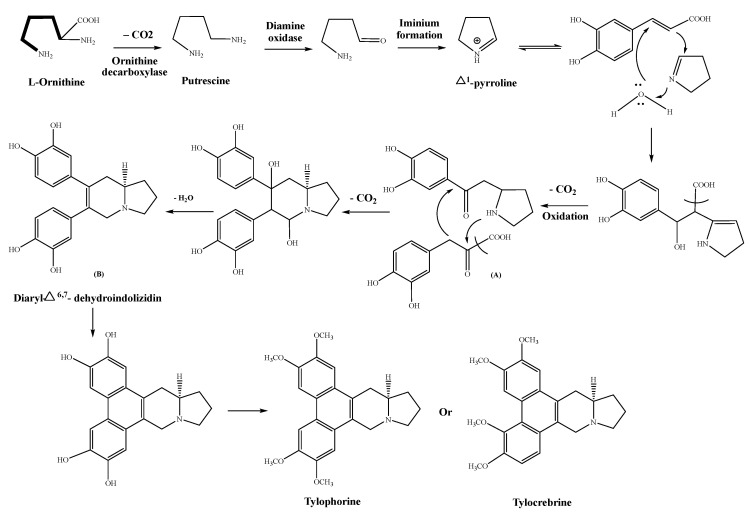
Biogenetic outline of the genus *Tylophora* alkaloid.

Phenanthroindolizidine alkaloids are a small group of naturally occurring compounds isolated from the genera *Tylophora, Pergularia,* and *Cynanchum* of the family Asclepiadaceae. A detailed biogenetic pathway is outlined in Figure 1. The biogenesis of these pentacyclic phenanthroindolizidine alkaloids, e.g., tylophorine, and other structurally inter-related compounds containing four to five ring units, is derived from different amino acid (AA)-based precursors, such as tyrosine, phenylalanine, and ornithine [85,86]. The later AA contains both α- and δ-amino groups, and the nitrogen of the previous group is involved with the carbon chain in the formation of the alkaloidal structure barring its carboxyl group. In this pathway, ornithine supplies a C_4_N structural block, basically a pyrrolidine ring, for advancing biogenesis of the alkaloid. The reactivity of ornithine is nearly matched by L-lysine, which manipulates a C_5_N unit containing its amino group towards the formation of the molecule [77]. Mechanistically, the pyrrolidine ring system is originally formed as a Δ^1^-pyrrolinium cation, and the putrescine, along with oxidative deamination by the action of a diamine oxidase, produces the required aldehyde. The Δ^1^-pyrrolinium cation is further transformed to imine, and in the presence of water, upon the involvement of cinnamic acid, it forms the emerging skeleton of the developing alkaloid. Ring B of an alkaloidal structure is formed by tyrosine, while ring A is formed by phenylalanine. Phenylalanine is consolidated through cinnamic, caffeic, and *p*-coumaric acids to produce the alkaloids’ structures. Owing to further modifications in the biogenetic pathways, a convenient hydroxylation pattern develops with the participation of *p*-coumaric, or caffeic acid. The important steps of oxidation and decarboxylation, and the condensation of 3-hydroxyphenylpyruvic acid followed by transformation of the carbinol amine, result in the formation of a diaryl-7-dehydroindolizidine intermediate, which is a seco structure. Finally, phenol oxidative coupling results in the formation of tylophorine and tylocrebrine structures via position 2 and 6 couplings, respectively. The involvement of methionine completes the methylation step(s) of the OH group(s) of the final alkaloid structure [85,86,87].

## 6. Biotechnical Production of Tylophorine

### 6.1. Production of Tylophorine and Agrobacterium-Mediated Transformation

*Agrobacterium rhizogenes*, a Gram-negative bacterium that is mainly located in the soil, causes infection in plants [88]. It transfers T-DNA, a 25-base pair oligonucleotide replication through a transformation procedure from roots, presenting plasmid (Ri) to the influenced plant’s genome [89]. During this transformation, hairy roots are produced at the spot of infection. This technique is considered one of the most effective pathways for manufacturing required secondary metabolic compounds, without causing any damage to the original plant, and with continuous production of the desired secondary metabolites within a short period of time. The transgenic root production technique has been standardized in *T. indica* after infection of its aerial parts and intact shoots by *Agrobacterium rhizogenes* (LBA 9402 and A4 strains). The roots and calli were prompted at different locations [90,91]. The response was a result of several underlying factors, such as the type of strains (Gram positive and Gram negative), and explants used, as well as the site of infection. The A4 strain was the only one that recorded a response inducing the transformation process. The maximum rate of transformation was reported to be around 60% with the intact shoots confirmed by PCR analysis. The production of tylophorine (its structure is shown in Figure 1) from different root clones was variable, and the maximum root biomass and tylophorine were obtained in about one month of suspension culture. The roots were dried, powdered, and subjected to defatting with a non-polar solvent for 24 h, followed by shaking with chloroform for a similar period of time. The extracts were pooled together, dried by evaporation, and the residue was re-extracted with chloroform three times, and separated by a separating funnel. The extracted portions were combined and dried, then filtered using a Millipore^®^ filter (0.2 mm). An analysis using HPLC (High-Performance Liquid Chromatography) was performed to obtain the maximum tylophorine yield of 1.29 ± 0.5 mg/g DW (dry weight) [92].

### 6.2. Extraction from Suspension Cultures Callus, and Dried Leaves

A quantitative analysis using HPTLC (High-Performance Thin Layer Liquid Chromatography) technique was performed to quantify and extract tylophorine (its structure is shown in Figure 1) from *Tylophora* spp. dried ground leaves, callus, and suspension cultures [93]. The technique depended on extraction with methanol acidified with acetic acid, followed by EtOAc (ethyl acetate) extraction. By using the Rf values of test samples to compare with the Rf value of the reference standard sample, the material’s presence was quantified. Variability of tylophorine concentration in the three samples, obtained from leaves extract, leaf-based callus, and suspension extract was quantified. Leaf extract attained the maximum level of tylophorine in the sample, followed by the leaf callus, and the suspension extract, with 80, 24.46, and 28.30 μg/mL, respectively.

Another study was performed to determine the tylophorine contents in the leaves of *T. indica* [94]. Dried and powdered leaves were soaked with *n*-hexane to eliminate the non-polar constituents. After that, they were macerated with EtOAc at pH 3.5–4, adjusted by hydrochloric acid. The extract was diluted with distilled water, evaporated to 50% of its volume at 60 °C by an evaporator, and washed with dichloromethane three times. NaOH was used to adjust the pH between 11–13, and the extract was again concentrated, and HPTLC analysis was performed at a wavelength of 258 nm. The quantification of the tylophorine was calculated using the following formula:

Concentration μg/mL= Area of peak of standard in test sample×Concentration of standardArea of standard peak

## 7. Biological Activities

### 7.1. Biological Activities of Genus Tylophora

**Figure 2:**
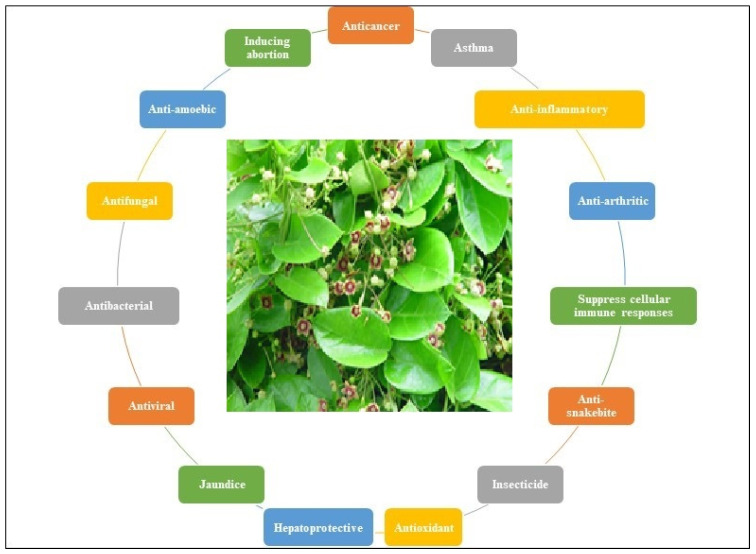
Important biological activities of the genus *Tylophora* (The plant picture is for *T. indica*).

*Tylophora* species are pharmacologically active. Several species of the genus *Tylophora* have been associated with diverse biological actions. The medicinal properties of some of the plants belonging to the genus *Tylophora* (Table 5 and Figure 2), and their bioactive constituents, are presented in Table 6.

### 7.2. Structure-Activity Relationship (SAR) of Tylophorine

Tylophorine (its structure is shown in Figure 1), the naturally abundant phenanthroindolizidine alkaloid, studied for its potential to inhibit cancer cell growth, has shed light on its structure-activity relationships (SAR) Previous studies on the phenanthroindolizidine alkaloids showed that a rigid phenanthrene ring is necessary for strong cytotoxicity. The absence of an indolizidine ring, or the presence of an OMe (ethereal methyl) group at position 2, results in a loss of cytotoxicity [132]. It was also believed to exert its anti-cancer effects through modulation of the vascular endothelial growth factor receptor (VEGFR2). VEGFR2 plays a crucial role in regulating cell growth, cell survival, cell proliferation, and the cells’ overactivation, which is a hallmark of many types of cancer. Tylophorine was thought to disrupt signaling pathways that cause cancer cell growth and survival by modulating VEGFR2 receptors, resulting in decreased cancer cell proliferation and increased cell death. Studies have shown that tylophorine binds to certain VEGFR2 receptors and modulates their activity, leading to the inhibition of cancer cell growth. The interaction between tylophorine and VEGFR2 was found to have a stable conformation based on in silico analysis. The results showed that hydrogen bonding and aromatic interactions were involved [129].

## 8. Clinical Trials on Genus *Tylophora* and Its Compounds

In some clinical trials, *Tylophora* extracts, and/or their active compounds, e.g., tylophorine (its structure is shown in Figure 1), have been evaluated for their efficacy, and safety as a therapeutic agent. Double-blind studies were performed on *Tylophora indica* for the treatment of both asthma and allergic rhinitis. However, further investigation is needed to understand the potential benefits and limitations of *Tylophora*-based therapies. Prospective clinical trials with a larger sample size and well-designed protocols are required to validate the therapeutic efficacy of *Tylophora* [96,133,134].

## 9. Cultivation Potential of *Tylophora indica*

*Tylophora* cultivation on a commercial scale is becoming increasingly popular due to its abundant medicinal properties. However, its cultivation is still in its infancy and early stages, and is limited to only a few regions. The plant prefers well-drained soil, moderate to high humidity, and shade. It can be propagated from seeds or cuttings. *Tylophora* plants grow best in regions with a tropical or subtropical climate, but can also be grown in greenhouses. Adequate moisture, well-drained soil, and proper temperature control are crucial for its growth, propagation, and survival [13]. To promote the cultivation of *Tylophora* genus plants, further horticultural work is needed to improve the growth, propagation, and yield of the plants, which includes identifying and controlling the factors and conditions responsible for optimal growth, developing new cultivation techniques, and increasing the availability of high-quality planting materials.

## 10. Commercial Potential of Genus *Tylophora*

The industrial potential of *Tylophora* lies in high-yielding plant varieties, alkaloid extraction and isolation, and the efficient separation of bioactive compounds from extracts and enriched materials. Their use in the pharmaceutical and health food industries is important. Tylophorine (its structure is shown in Figure 1) is the major isolate in both *Tylophora indica* and *Tylophora asthmatica* plants, and has shown promise as a potential drug candidate in preclinical studies. The compound can be developed into a new drug to treat cancer types, and other diseases based on its pharmacologically confirmed and traditionally consistent and beneficial medicinal uses. In addition, the extracts of *Tylophora* species are used in cosmetic and personal care products for their moisturizing, anti-inflammatory, and antioxidant properties. The high demand for natural and safe cosmetic ingredients has created a market opportunity for *Tylophora* extracts as well. Nonetheless, *Tylophora* has significant potential as a source of bioactive compounds for the pharmaceutical, health, food, and cosmetic industries. Further product development efforts are needed to fully exploit its potential and bring its benefits to a wider public.

*Tylophora indica* powder, also known as Indian ipecacuanha, is used to treat allergies, asthma, congestion, constipation, cough, cancer, inflamed skin, diarrhea, bloody diarrhea, hemorrhoids, gas, gout, liver disorders, jaundice, joint pain, symptomatic relief for rheumatoid arthritis, and whooping cough, as well as to induce vomiting, and cause sweating. The herb is part of the ancient Indian system of medicine, Ayurveda, and the currently practiced Ayush system. The herb’s mother tincture as a homeopathic drug is also available on the market. The *Tylophora indica* herb’s fresh leaves are chewed and swallowed on daily basis for a week with water in the early morning for emptying the stomach. Its use also provides partial or full relief from asthma. The root powder of the herb is used for diarrhea, dysentery, and intermittent malarial fever. However, the user’s age and health conditions must be considered before any medicinal use of the herb [135,136].

## 11. Conclusions

Plants from the genus *Tylophora* are widely distributed in the tropical and subtropical regions of warm and wet climatic southern-hemisphere countries. The plants are medicinally viable species that have been documented in various anthropological societies for their traditional uses against various physiological and hormonal disorders. Activities such as anti-cancer, anti-tumor, broad-spectrum anti-microbial, anti-fungal, and anti-virus activities have been reported and pharmacologically established for plants of the genus. Other pharmacological activity confirmation though symptomatic treatment of physiological disorders is imperative. Molecular modeling-based activity predictions of the nearly forty-four phenanthroindolizidine alkaloids, which are abundant in eight plant species, are tasks for the future. There is still a pressing need to pursue extensive phytochemical screening and bioassay-guided activity confirmations using the extracts, and subsequent and designated fractions, as well as determining the biological activity of isolated pure constituents of known, novel, and new structures, especially of an alkaloidal nature, towards finding new bioactive chemical entities and molecular templates for oncological and other aspects of drug design and discovery.

## Figures and Tables

**Table 1:** List of accepted species in the genus *Tylophora* and their synonyms.

Plant Species	Synonym
*Tylophora anomala* (NE Br.)	
*Tylophora anthopotamica* (Hand-Mazz.)	
*Tylophora apiculata* (Schum K.)	
*Tylophora arachnoidea* (Goyder)	
*Tylophora arenicola* (Merr.)	
*Tylophora astephanoides* (Tsiang-PT Li)	
*Tylophora asthmatica* (Wight Lf)	
*Tylophora augustiniana* W Craib (Hemsl.)	
*Tylophora auriculata* (Makino)	*Vincetoxicum sublanceolatum* vr. *auriculata* (Franch.-Sav)
*Tylophora badia* (E. Mey.) Schltr.	*Tylophora badia* vr. *badia*
*Tylophora brevipes* (Turcz.) Fern.-Vill.	
*Tylophora brownii* (Hayata)	*Tylophora ovata* vr. *brownii* Tsiang & P.T. Li (Hayata)
*Tylophora cameroonica* (NE Br.)	
*Tylophora chingtungensis* (Tsiang-PT Li)	
*Tylophora cinerascens* P Forst (R. Br.)	*Marsdenia cinerascens* (R Br) *Pergularia cinerascens* Spreng (R Br)
*Tylophora coddii* (Bulloc)	
*Tylophora congoensis* (Schltr)	
*Tylophora congolana* (Baill, Bulloc)	
*Tylophora conspicua* (NE Br.)	
*Tylophora cordata* (Druce)	*Tylophora syringaefolia* (E Mey)
*Tylophora costantiniana* MG. Gilbert, WD. Stevens (Tsiang)	
*Tylophora cycleoides* (Tsiang)	
*Tylophora dahomensis* (K Schum)	
*Tylophora dickinsii* Makino (Franch. & Sav.)	*Vincetoxicum sublanceolatum* var. dickinsii Franch. & Sav.
*Tylophora erubescens* (Meve & Liede)	
*Tylophora flanaganii* (Schltr)	
*Tylophora fleckii* (Schltr, NE Br.)	*Tylophoropsis fleckii* (Schltr)
*Tylophora flexuosa* RBr.	*Hoya flexuosa* (R. Br.) Spreng., *Tylophora carnosa* (Wal ex Wight.), *Tylophora tenuissima* (Roxb, Wight-Arn), *Tylophora tetrapetala* (Dennst-Suresh), *Vincetoxicum flexuosum* (R. Br-Kuntze), *Tylophora dielsii* (H Lév Hu.), *Tylophora tenuis* (Blume)
*Tylophora floribunda* (Miq)	*Tylophora chungii* (Merr), *Vincetoxicum floribundum* (Miq; Franch, Sav), *Tylophora shikokiana* (Matsumone)
*Tylophora forrestii* ME. (Gilbert-PT-Li)	
*Tylophora gilletii* (Wild)	
*Tylophora glabra* (Costantin)	*Tylophora renchangii* (Tsiang), *Tylophora renchangii* (Tsiang)
*Tylophora glabriflora* (Warb, Schltr)	
*Tylophora glauca* (Bullock)	
*Tylophora govanii* (Wight-Arn) Decne.	
*Tylophora gracilenta* (Tsiang-PT Li)	
*Tylophora gracilis* (Wild)	
*Tylophora gracillima* (Mark.)	
*Tylophora henryi* (Warb)	
*Tylophora heterophylla* (Rich A.)	
*Tylophora hirsuta* (Wight)	*Diplolepis apiculata* (Lindl)
*Tylophora hui* (Tsiang)	
*Tylophora indica* (Burm.f.-Merr.)	
*Tylophora inhambanensis* (Schltr)	
*Tylophora insulana* (Tsiang-PT Li)	
*Tylophora iringensis* (Goyder-Markgr)	*Pentarrhinum iringense* (Markgr)
*Tylophora javanica* (Boerl-Hassk)	*Hybanthera javanica* (Hassk)
*Tylophora kerrii* (Craib WG)	*Tylophora balansae* (Costantin)
*Tylophora koi* (Mer)	*Tylophora taiwanensis* (Hatus), *Tylophora sootepensis* (Craib WG)
*Tylophora leptantha* (Tsiang)	
*Tylophora longifolia* (Wight)	
*Tylophora longipedunculata* (Schweinfurth-Hoehnel, Schltr)	*Gymnema longepedunculatum* Schweinfurth-Hoehnel, *Sphaerocodon longipedunculatum* (Schweinfurth-Hoehnel) K. Schum.
*Tylophora lugardae* (Bullock)	
*Tylophora lycioides* (Decne, E Mey.)	
*Tylophora membranacea* (Tsiang-PT Li)	
*Tylophora merrillii* (Schltr)	
*Tylophora multiflora* (Alston, Wight-Arn)	*Iphisia multiflora* Wight & Arn.
*Tylophora nana* (Schneid CK)	*Tylophora nana* vr. gansuensis (Wang LC-Sun XG)
*Tylophora nikoensis* Matsum (Franch-Sav)	*Vincetoxicum nikoense* (Franch-Sav)
*Tylophora oblonga* NE Br.	*Tylophora liberica* NE Br., *Tylophora anfracta* NE.Br.
*Tylophora obtusula* (Tanaka ex Franchet & Savatier) Makino	*Vincetoxicum sublanceolatum* vr. obtusulum (Tanaka)
*Tylophora oculata* NE Br.	
*Tylophora oligophylla* (Tsiang-Gilbert MG., Stevens WD.)	
*Tylophora* oshimae (Hayata)	
*Tylophora ovata* (Hook-Lindl.)	*Tylophora atrofolliculata* F.P. Metcalf, *Tylophora hispida* vr. (*brownie*-Hayata), *Tylophora jacquemontii* (Decne), *Tylophora lanyuensis* Lu & Liu, *Tylophora hispida* (Decne), *Tylophora mollissima* (Wall), *Tylophora panzhutenga* (Zhu ZY), *Tylophora ovata* vr. *ovata*
*Tylophora picta* Tsiang	
*Tylophora* plagiopetala (Schum K. & Schltr)	
*Tylophora rockii* (PT Li & Gilbert MG)	
*Tylophora rotundifolia* (Ham-Buch.)	*Tylophora trichophylla* (Tsiang)
*Tylophora secamonoides* (Tsiang)	
*Tylophora silvestrii* (Tsiang-PT Li, Pamp.)	*Henrya silvestrii* (Pamp.) *Henryastrum silvestrii* (Happ-Pamp.)
*Tylophora simiana* (Schltr)	
*Tylophora stenoloba* (NE Br.-Schum K)	
*Tylophora subnuda* (Sm. AC-Gray A)	
*Tylophora sylvatica* (Decne)	*Tylophora bojeriana* (Decne) *Vincetoxicum sylvaticum* (Kuntze, Decne)
*Tylophora tenerrima* (Wight)	
*Tylophora tengii* (Tsiang)	
*Tylophora tenuipedunculata* (Schum K.)	
*Tylophora tsiangii* (PT Li & Gilbert MG)	*Vincetoxicum tsiangii* (PT Li)
*Tylophora tuberculata* (PT Li & Gilbert MG)	
*Tylophora umbellata* (Schltr)	
*Tylophora uncinata* (PT Li & Gilbert MG)	
*Tylophora urceolata* (Meve)	
*Tylophora velutina* G. Don (R Br.)	*Pergularia velutina* (R Br.) Spreng, *Marsdenia velutina* R Br.
*Tylophora yunnanensis* (Schltr)	
*Tylophora zenkeri* (Schltr)	

**Table 2:** Ethno-medicinal uses of genus *Tylophora* species.

*Tylophora* Species	Ethno-Medicinal Applications	Location	Reference
*T. asthinatica*	Treatment of bronchial asthma and allergy	India, Pakistan, and Indonesia	[54,55]
*T. villosa*	Treatment of liver disorders	Indonesia	[56]
*T. hirsuta*	Treatment of diabetes, treatment of eye diseases in veterinary medicine	India and Pakistan	[57,58,59]
*T. indica*	Treatment of asthma, dermatitis, constipation (flower part), dysentery, cough, snake poison, and rheumatic conditions. The plant is also used as an expectorant, diaphoretic, and emetic agent.	Bangladesh, India (Orissa state)	[60,61,62,63,64]
*T. fasciculata*	Treatment of fever and body pain	Orissa	[62]
*T. atrofolliculata*	Treatment of rheumatoid arthritis	China	[65,66,67,68]
*T. ovata*	Treatment of rheumatism, asthma, and traumatic injury	China, and Taiwan	[42]
*T. floribunda*	Treatments of irregular menses	Chania and Hong Kong	[69]
*T. barbata*	Treatments of inflammation	Australia	[69]
*T. perrottetiana*	Treatments of wounds	Sri Lanka	[70]

**Table 3:** Phenanthroindolizidine alkaloids isolated from the genus *Tylophora*.

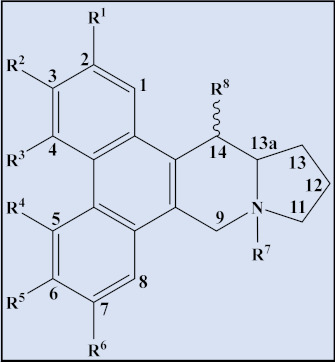																
Cpd	Trivial Name	Plant Name	R^1^	R^2^	R^3^	R^4^	R^5^		R^6^	R^7^	R^8^		13a		Reference	
1	Isotylocrebrine	*T. tanakae* *T. indica*	H	OCH_3_	OCH_3_	H	OCH_3_		OCH_3_	-	-		*S*		[73,74,75]	
2	Tylophorine	*T. tanaka,* *T. indica* *T. crebriflora*	OCH_3_	OCH_3_	H	H	OCH_3_		OCH_3_	-	-		*R*		[73,74,75,76]	
3	3-dimethyl isotylocrebrine	*T.tanakae*	H	OH	OCH_3_	H	OCH_3_		OCH_3_	-	-		*S*		[73,74]	
4	3-demethyl tylophorinidine	*T. atrofolliculata*	H	OH	H	H	OH		OCH_3_	-	α-OH		*S*		[65]	
5	3-demethyl-14α-hydroxy isotylocrebrine	*T. tanakae*	H	OH	OCH_3_	H	OCH_3_		OCH_3_	-	α-OH		*S*		[73,74]	
6	Isotylocrebrine *N*-Oxide	*T. tanakae*	H	OCH_3_	OCH_3_	H	OCH_3_		OCH_3_	O	-		*S*		[73,74]	
7	14α-hydroxy isotylocrebrine *N*-Oxide	*T. tanakae*	H	OCH_3_	OCH_3_	H	OCH_3_		OCH_3_	O	α-OH		*S*		[73,74]	
8	3-demethyl-14α-hydroxy isotylocrebrine *N*-Oxide	*T. tanakae*	H	OH	OCH_3_	H	OCH_3_		OCH_3_	O	α-OH		*S*		[73,74]	
9	6-demethyltylocrebrine	*T. tanakae*	OCH_3_	OCH_3_	H	OCH_3_	OH		H	-	-		*S*		[73,74]	
10	Tylophorinine	*T. indica* and *T. asthmatica*	H	OCH_3_	H	H	OCH_3_		OCH_3_	-	α-OH		*S*		[75,77,78]	
11	Tylophorinine *N*-Oxide	*T. tanakae* and *T. atrofolliculata*	H	OCH_3_	H	H	OCH_3_		OCH_3_	O	α-OH		*S*		[66,73,74]	
12	7-demethyltylophorine	*T. tanakae*	OCH_3_	OCH_3_	H	H	OCH_3_		OH	-	-		*R*		[73,74]	
13	Tylophorine *N*-Oxide	*T. tanakae*	OCH_3_	OCH_3_	H	H	OCH_3_		OCH_3_	O	-		*R*		[73,74]	
14	7-demethyltylophorine-N-Oxide	*T. tanakae*	OCH_3_	OCH_3_	H	H	OCH_3_		OH	O	-		*R*		[73,74]	
15	Antofine	*T. crebriflora*	OCH_3_	OCH_3_	H	H	OCH_3_		H	-	-		*R*		[76,79]	
16	14α-hydroxy isotylocrebrine	*T. indica* and *T. hirsuta*	H	OCH_3_	OCH_3_	H	OCH_3_		OCH_3_	-	α-OH		*S*		[78,80]	
17	4-demethyl isotylocrebrine	*T. hirsuta*	H	OCH_3_	OH	H	OCH_3_		OCH_3_	-	-		*S*		[80]	
18	3,6 Didemethyl isotylocrebrine	*T. tanakae*	H	OH	OCH_3_	H	OH		OCH_3_	-	-		α-OH		[73]	
19	14-α-Hydroxy 3,6 didemethyl isotylocrebrine	*T. tanakae*	H	OH	OCH_3_	H	OH		OCH_3_	-	α-OH		α-OH		[73]	
20	Tylocrebrine	*T. indica* and *T crebriflora*	OCH_3_	OCH_3_	H	OCH_3_	OCH_3_		H	-	-		*S*		[75,76]	
21	Tylophorinidine	*T. indica* and *T. atrofolliculata*	H	OCH_3_	H	H	OH		OCH_3_	-	α-OH		*S*		[66,75]	
22	5-hydroxy-*O*-methyltylophorinidine	*T. indica* and *T. hirsuta*	H	OCH_3_	H	OH	OCH_3_		OCH_3_	-	α-OH		*S*		[78,80]	
23	Tylophorinidine *N*-Oxide	*T. atrofolliculata*	H	OCH_3_	H	H	OH		OCH_3_	O	α-OH		*S*		[66]	
24	13 α-Hydroxytylophorine	*T. hirsuta*	OCH_3_	OCH_3_	H	H	OCH_3_		OCH_3_	-	-		*R*		[81]	
25	6-demethyltylophorine	*T.* *indica*	OCH_3_	OCH_3_	H	H	OH		OCH_3_	-	-		*R*		[78]	
26	4&6-desmethyl isotylocrebrine	*T.* *indica*	H	OCH_3_	OH	H	OH		OCH_3_	-	-		*S*		[78]	
27	Tyloindicine A	*T.* *indica*	H	OCH_3_	OCH_3_	OCH_3_	OCH_3_		H	-	-		*S*		[78]	
28	Tyloindicine D	*T.* *indica*	H	OCH_3_	OCH_3_	OH	OCH_3_		OCH_3_	-	-		*S*		[78]	
29	Tyloindicine E	*T.* *indica*	H	OCH_3_	H	H	OH		OCH_3_	-	-		*S*		[78]	
30	Cryptopleurine	*T. crebriflora*	OCH_3_	OCH_3_	H	H	OCH_3_		H	-	-		*R*		[76]	
31	Tylophorinicine	*T.* *asthmatica*	OCH_3_	OCH_3_	H	H	OCH_3_		OCH_3_	-	β-OH		*R*		[82]	
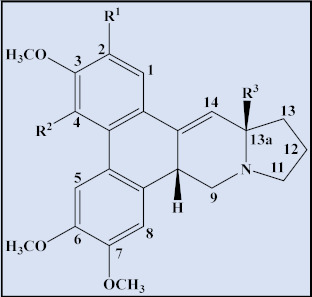																
**Compound**	**Trivial name**	**Plant name**	**R^1^**			**R^2^**				**R^3^**						**Reference**
32	Tyloindicine G	*T.* *indica*	OCH_3_			H				OH						[83]
33	Tyloindicine H	*T.* *indica*	H			OH				H						[83]
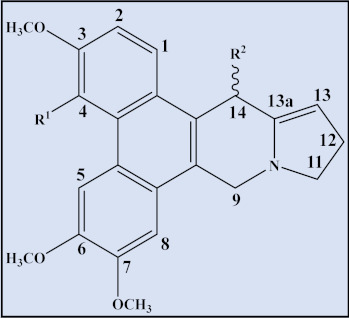																
**Compound**	**Trivial name**	**Plant name**	**R^1^**				**R^2^**									**Reference**
34	Tylohirsutinine	*T. hirsuta*	OCH_3_				H									[84]
35	Tylohirsutinidine	*T. hirsuta*	OH				αOH									[84]
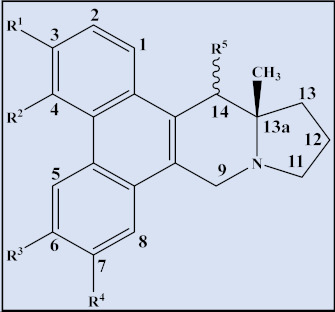																
**Compound**	**Trivial name**	**Plant name**	**R^1^**		**R^2^**		**R^3^**			**R^4^**				**R^5^**		**Reference**
36	Tyloindicine C	*T. indica*	OCH_3_		OH		OH			OCH_3_				-		[78]
37	13a-Methyltlohirsutine	*T. hirsuta*	OCH_3_		OCH_3_		OCH_3_			OCH_3_				-		[84]
38	13a-Methyltyl-ohirsutinidine	*T. hirsuta*	OCH_3_		OH		OCH_3_			OCH_3_				α-OH		[84]
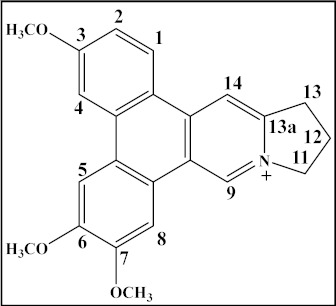	Tylophoridicine D	*T. atrofolliculata*														[66]
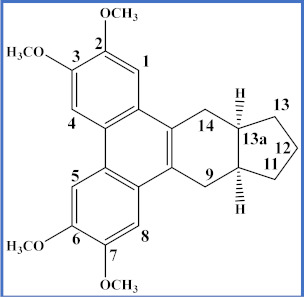	Tyloindane	*T. indica*														[83]

**Table 4:** Secophenanthroindolizidine alkaloids isolated from the genus *Tylophora*.

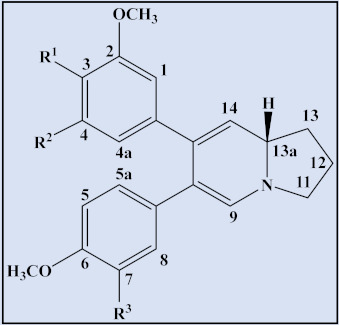							
Compound	Trivial Name	Plant Name	R^1^	R^2^		R^3^	Reference
1	Tyloindicine I	*T. indica*	OCH_3_	OH		OCH_3_	[83]
2	Tyloindicine J	*T. indica*	H	H		OCOCH_3_	[83]
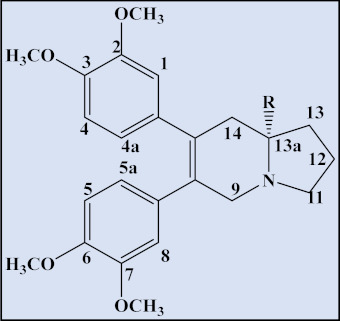							
Compound	Trivial name	Plant name	R				Reference
3	Septicine	*T. Tanakae* and *T. indica*	H				[73,75]
4	13a-Hydroxysepticine	*T. hirsuta*	OH				[84]
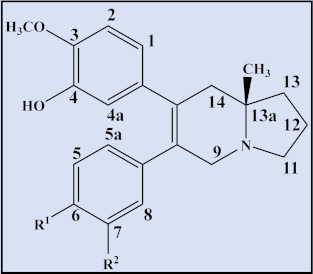							
Compound	Trivial name	Plant name	R^1^		R^2^		Reference
5	Tylohirsuticine	*T. hirsuta*	OCH_3_		OCH_3_		[80]
6	Tyloindicine B	*T. indica*	H		OCOCH_3_		[78]
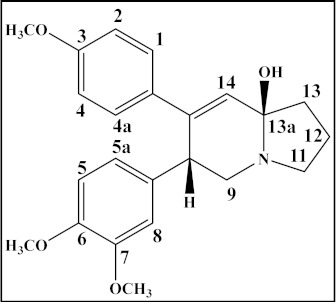	Tyloindicine F	*T. indica*					[83]

**Table 5:** Uses and biological activities of various *Tylophora* species.

Plant Species	Uses/Biological Properties	Reference(s)
*Tylophora indica*	Asthma	[82,83,84,85,86,87,95,96]
	Anti-inflammatory, and anti-arthritic	[95]
	Suppress cellular immune responses	[97,98]
	Anti-snake-bite	[99]
	Anti-viral	[100]
	Antitumor	[14,25,101,102]
	Insecticide	[103]
	Free radical scavenging activity	[32,104,105,106]
	Hepato-protective activity	[22,107,108,109,110,111]
	Anti-bacterial	[36,53,112,113,114,115]
*Tylophora asthmatica*	Asthma and dry cough	[116,117]
	Anti-snake-bite	[118]
	Jaundice/Liver protective	[116]
	Anti-bacterial and Anti-fungal activity	[119]
*Tylophora hirsute*	Abortifacients (abortion inducer)	[120]
*Tylophora floribunda*	Anti-cancer	[121]
*Tylophora tanakae*	Cytotoxic, Insecticide	[72,73]
*Tylophora dalzellii*	Anti-tumor	[102]
*Tylophora atrofolliculata*	Anti-tumor	[122]
*Tylophora pauciflora*	Anti-tumor, anti-viral, anti-fungal, anti-bacterial, anti-amoebic activities, anti-inflammatory, and antibiotic; insecticidal	[49,123]

**Table 6:** Biological activities of alkaloidal constituents isolated from plants of the genus *Tylophora*.

Active Constituent	*Tylophora* Species	Uses	Reference
Tylophorine (its structure is shown in Figure 1)	*Tylophora indica*	Cytotoxic activity	[124]
		Insecticidal activity	[124]
		Anti-inflammatory	[42]
		Stimulant to adrenal Cortex	[54]
		Anti-feedant activity	[53]
		Anti-viral activities	[125,126,127]
		Anti-bacterial	[53]
		Anti-amoebic	[81]
		Anti-fungal	[53]
		Hepato-protective activity	[22]
		Anti-allergic activity	[97,128]
		Anti-angiogenic	[129]
		Diuretic activity	[130]
		Inhibition of cellular immune responses	[97]
		Arthritis	[131]
	*Tylophora asthmatica*	Asthma	[116,117]
Tylophorinicine	*Tylophora indica*	Cytotoxic activity Insecticidal activity	[116,117,130,131]
	*Tylophora asthmatica*	Asthma	
Tylophorinine	*Tylophora indica*	Cytotoxic activity Insecticidal activity	[124]
	*Tylophora atrofolliculata*	Cytotoxic activity on HCT-8 cell	[66]
Tylophorinine-*N*-Oxide	*Tylophora indica*	Cytotoxic activity Insecticidal activity	[124]
Tylophorinidine	*Tylophora indica*	Cytotoxic activity Insecticidal activity	[124]
	*Tylophora atrofolliculata*	Cytotoxic activity on HCT-8 cell	[66]
Tylophorinidine-*N*-Oxide	*Tylophora indica*	Cytotoxic activity Insecticidal activity	[124]
3-Demethyltylophorinidine			
Septicine	*Tylophora indica*		
3,6-Didemethyl isotylocrebrine	*Tylophora tanakae*	Cytotoxic activity	[73]
14-α-Hydroxy 3,6 didemethyl isotylocrebrine			
Tylophoridicines C-F	*Tylophora atrofolliculata*	Cytotoxic activity on HCT-8 cell	[66]
R-(+)-Deoxytylophorinidine	*Tylophora atrofolliculata*		

## Data Availability

Not applicable.
